# Hemoglobin outcomes associated with supervised therapeutic iron or preventive multiple micronutrient powder supplementation in high-altitude Peruvian infants: A real-world natural experiment

**DOI:** 10.1371/journal.pone.0356793

**Published:** 2026-08-26

**Authors:** Cinthya Vásquez-Velásquez, Parminder S. Suchdev, Chris A. Rees, Yury R. Quispe-Lipa, Lidia S. Caballero, Gustavo F. Gonzales

**Affiliations:** 1 Carrera de Ingeniería Biomédica, Facultad de Ingeniería e Inteligencia Artificial, Universidad San Ignacio de Loyola, Lima, Peru; 2 Hubert Department of Global Health, Rollins School of Public Health, Emory University, Atlanta, Georgia, United States of America; 3 Department of Pediatrics, Emory University, Atlanta, Georgia, United States of America; 4 Division of Pediatric Emergency Medicine, Emory University School of Medicine, Atlanta, Georgia, United States of America; 5 Department of Emergency Medicine, Children’s Healthcare of Atlanta, Atlanta, Georgia, United States of America; 6 Escuela Profesional de Nutrición Humana, Universidad Nacional del Altiplano, Puno, Peru; 7 Carrera de Medicina Humana, Facultad de Ciencias de la Salud, Universidad San Ignacio de Loyola, Lima, Peru; Kintampo Health Research Centre, GHANA

## Abstract

**Background:**

In Peru, legally mandated universal micronutrient supplementation policies preclude randomized trials.

**Objective:**

To evaluate hemoglobin (Hb) outcomes associated with enhanced follow-up of therapeutic iron or preventive multiple micronutrient powder (MNP) supplementation among high-altitude infants within a real-world natural experiment.

**Methods:**

This observational study leveraged a policy-driven natural experiment to analyze two population-based cohorts of 317 infants aged 6–12 months from Puno, Peru (3,832 m), evaluated before and after implementation of strict follow-up. According to national guidelines, infants with anemia received therapeutic iron syrup (ferrous sulfate or iron polymaltose), while non-anemic infants received preventive MNP. Supplements were provided monthly for six months. Hemoglobin was measured at baseline and follow-up and adjusted for altitude using the WHO 2024 criteria. Adequate Hb response was defined as an increase ≥1 g/dL. Multivariable regression models assessed factors associated with hemoglobin response.

**Results:**

Overall, 14.8% of infants achieved an Hb increase ≥1 g/dL (18.7% vs. 11.8% in the strict and non-strict follow-up groups, respectively). Among non-anemic infants at baseline, adequate Hb response was more frequent under strict follow-up (8.3% vs. 2.7%). In the prespecified primary adjusted model, strict follow-up was associated with higher odds of achieving an adequate Hb response (aOR 3.53; 95% CI 1.40–8.84), compared with the crude estimate (OR 2.45; 95% CI 1.30–4.63). In sensitivity analyses incorporating additional socioeconomic, nutritional, and program-related covariates, the association was attenuated (aOR 2.18; 95% CI 0.53–8.99). When evaluated as a continuous outcome, strict follow-up was associated with greater hemoglobin increases (adjusted mean difference 0.74 g/dL; 95% CI 0.23–1.25). At six months, altitude-adjusted anemia prevalence was lower in the strict follow-up group than in the non-strict group (25.2% vs. 38.2%, p = 0.019). Baseline anemia and older age were independently associated with larger hemoglobin gains.

**Conclusions:**

Strict follow-up was associated with greater increases in hemoglobin concentration and lower altitude-adjusted anemia prevalence at follow-up. Although anemia prevalence was lower among infants receiving strict follow-up, residual anemia remained common after six months of supplementation. These findings suggest that enhanced programmatic follow-up may improve hematologic outcomes under routine public health conditions, while additional factors contributing to anemia persistence warrant further investigation.

## Introduction

Globally, anemia remains a major public health challenge despite decades of public health interventions such as iron supplementation and food fortification strategies [1,2]. Iron deficiency (ID) has traditionally been considered the leading cause of childhood anemia [1]; however, the limited population-level impact of iron interventions in many settings suggests that anemia etiology may be more heterogeneous than commonly assumed [3–5].

Hemoglobin (Hb) based definitions of anemia further complicate this issue. Since 1959, a single Hb cutoff of 11 g/dL has been used to define anemia for children aged 6–59 months, despite well-documented age-related variations in Hb during infancy [6,7]. In recognition of this limitation, the World Health Organization (WHO) revised its guidelines in 2024, lowering the anemia cutoff to 10.5 g/dL for infants aged 6–23 months [8].

These definitional challenges are particularly relevant in high-altitude (HA) populations, where physiological increases in Hb due to chronic hypoxia require altitude-specific adjustments to determine anemia.

Countries with a substantial proportion of their population living at HA have shown a limited response to universal iron supplementation programs aimed at reducing childhood anemia [9–11]. Peru, where approximately 30% of the population resides above 2,000 meters [12], mandates iron supplementations for all children aged 6–59 months [13]. Nevertheless, anemia prevalence in several HA regions remains extremely high, exceeding 60% despite long-standing iron supplementation programs [14].

In HA populations, physiological adaptation to hypoxia results in higher Hb concentrations, prompting WHO recommendations to adjust Hb values for altitude [8,15]. While these adjustments facilitate comparisons with sea-level populations, they also substantially influence estimated anemia prevalence. Several studies suggest that the high prevalence of anemia reported at HA may partly reflect limitations of altitude-adjusted Hb thresholds rather than anemia due to ID [16–20]. Supporting this interpretation, evidence indicates that body iron content and hepcidin levels may be higher at HA than at sea level, suggesting that iron stores may be adequate in these populations [16,21].

Chronic hypoxia may also influence iron metabolism beyond its effect on hemoglobin concentrations. Hypoxia regulates erythropoietic activity and modulates hepcidin, the main hormone involved in iron absorption and mobilization. Studies conducted in high-altitude populations suggest that these physiological adaptations may allow maintenance of adequate iron stores despite lower oxygen availability [22,48].

Consequently, infants who are not truly iron deficient may have a limited hematological response to preventive iron supplementation or MNP, which could partly explain the modest impact of universal supplementation programs observed in some high-altitude settings [23].

Recent WHO guideline updates further refined the interpretation of Hb concentrations at HA. In addition to lowering the anemia cutoff for young infants, the 2024 WHO guidelines revised altitude-adjustment factors, particularly above 3,500 meters, where correction values are now lower than earlier recommendations [8,15]. These changes substantially reduce estimated anemia prevalence compared with prior adjustment schemes, reinforcing concerns about the potential overestimation of anemia burden in these settings [5,24].

WHO guidelines recommend daily iron supplementation for infants and young children aged 6–23 months in settings with high anemia prevalence [25]. Although iron supplementation improves hematological indices in iron-deficient children, responses are attenuated in iron-replete infants, and some evidence suggests potential adverse effects on growth in this group [26]. These observations highlight the tight physiological regulation of iron metabolism and the potential risks of excess iron exposure [27], underscoring the need to critically evaluate both the effectiveness and implementation of universal supplementation strategies, particularly in physiologically distinct populations such as those living at HA.

In this context, multiple micronutrient powders (MNP) are recommended by the WHO as a preventive strategy for home or point-of-use fortification of complementary foods to reduce iron deficiency and anemia among infants and young children aged 6–23 months in populations with anemia prevalence above 20%. MNP are single-dose sachets containing iron, zinc, vitamin A, and other essential micronutrients that can be added to semi-solid foods without altering customary dietary practices, thereby improving adherence [28]. In contrast, infants diagnosed with anemia are managed with therapeutic iron formulations, such as ferrous sulfate or iron polymaltose, in accordance with national and international guidelines.

In Peru, randomized placebo-controlled trials of iron supplementation in infants are neither ethical nor legal, as supplementation is mandated by national policy. In 2017, the Ministry of Health (MoH) introduced a stricter, mandatory supervision framework for MNP supplementation in non-anemics and ferrous sulfate or iron polymaltose in anemics, incorporating structured monitoring and adherence support. This policy-driven change created a unique natural experiment, enabling comparison of hematological outcomes between two cohorts receiving identical supplementation regimens but differing in supervision intensity.

The objective of this study was to assess the association between policy-mandated strict supervision of MNP or therapeutic iron supplementation and Hb response and anemia outcomes among infants living at HA. We hypothesized that stricter supervision would improve adherence and increase the likelihood of a Hb response to MNP supplementation or therapeutic iron, but that it would have a limited effect on reducing overall anemia prevalence in this HA setting.

## Materials and methods

Study design: This study was a prospective observational cohort leveraging a policy-driven natural experiment. Infants receiving routine MNP supplementation or therapeutic iron were followed in the district of El Vallecito, Puno, Peru (3,832 m above sea level), between 01/08/2017 and 31/07/2019. The study was based on the district nominal registry, which captures all infants eligible for anemia screening and supplementation within the catchment area. Therefore, the study population closely represents the target population served by the local health system. The natural experiment resulted from the nationwide implementation of mandatory strict supervision for the anemia program in 2017, which enabled before–after comparison between cohorts exposed to identical supplementation regimens but differing in supervision intensity. In Peru, infant iron supplementation is mandated by law, precluding randomized or placebo-controlled trials.

In April 2017, the Ministry of Health issued Technical Health Standard RM N.° 250–2017-MINSA, establishing a structured supervision framework for anemia prevention and treatment. Although formally approved in 2017, implementation at the El Vallecito Health Facility was progressive. Full operationalization of the strict supervision framework, including home visits, active monitoring, and multidisciplinary follow-up, was achieved in September 2018. Because assignment to strict or non-strict follow-up depended solely on the timing of initiation of MNP or therapeutic iron supplementation relative to implementation of the supervision framework, this policy change created a natural experiment.

This phased rollout allowed comparison of two consecutive cohorts: a non-strict follow-up cohort (01/08/2017–31/08/2018) and a strict follow-up cohort (01/09/2018–31/07/2019). In both cohorts, Hb levels were measured at baseline (6–12 months of age) and after six months of supplementation (12–18 months).

The study was conducted at the Centro de Salud Vallecito, a level I-3 health facility located on the shores of Lake Titicaca in the Southern Peruvian Andes. The peri-urban population is predominantly Quechua and Aymara, and the facility serves approximately 400 children under three years of age annually.

All infants aged 6–12 months attending routine growth and development visits during the study period were eligible. Infants born outside Puno or with missing Hb data were excluded. Based on the initial sample of 320 infants, 0.94% were excluded due to inconsistent hemoglobin data, leaving a total of 317 infants, reflecting near-complete capture of the eligible population. Sample size was determined by exposure to each policy period, consistent with the natural experiment design.

**Fig 1:**
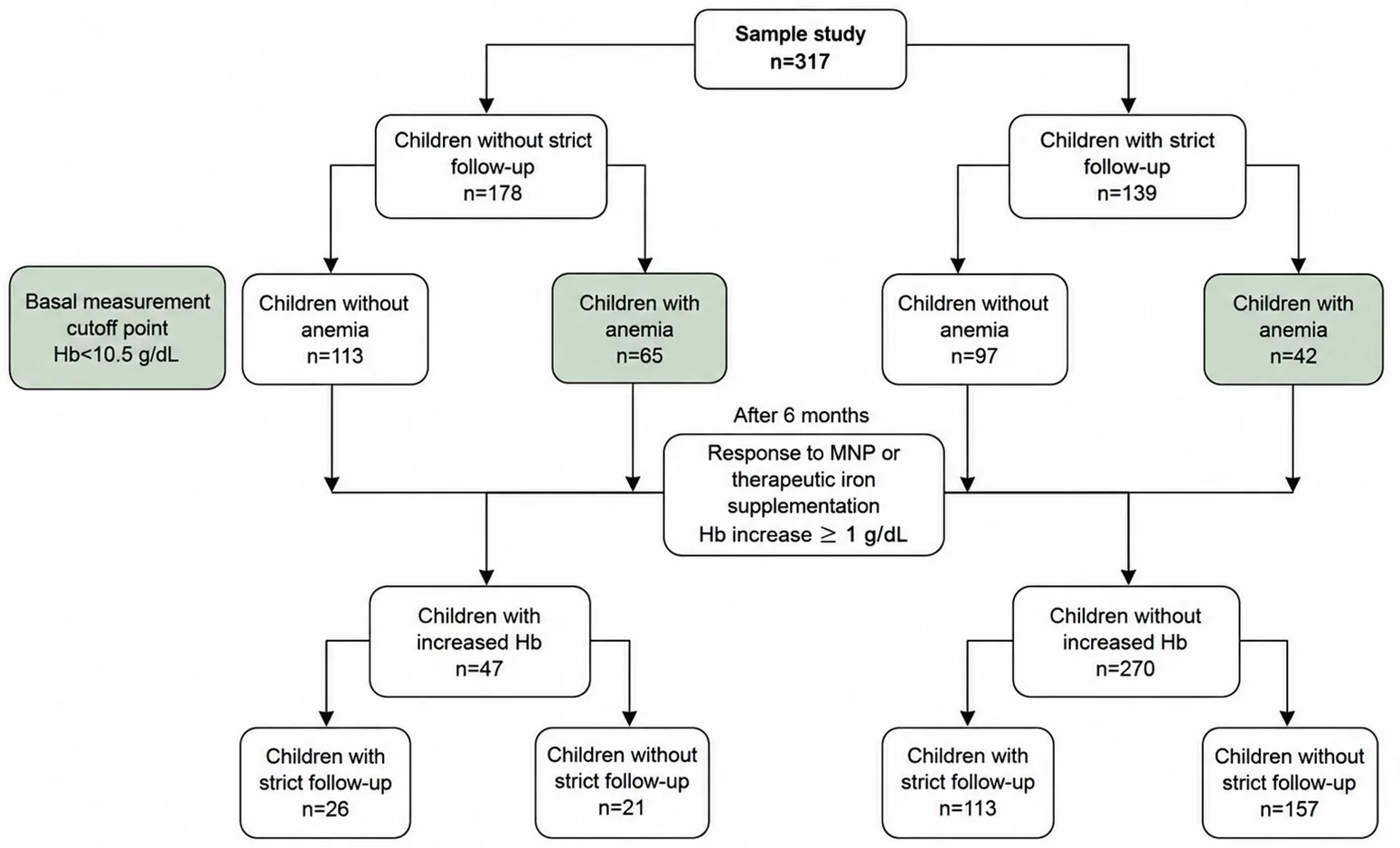
Flow chart of infants evaluated by group and follow-up performed. MNP: Multiple Micronutrient Powders. Hb: Hemoglobin.

**Groups and surveillance:** Infants were classified according to baseline anemia status (anemic or non-anemic based on Hb measured at the initial clinic visit) and analyzed by follow-up intensity (strict or non-strict) (Fig 1).

MNP was prescribed for six consecutive months in accordance with the 2017 guidelines of the Peruvian MoH, which are based on WHO recommendations [13] that differentiate iron intake recommendations according to anemia status. In children without anemia living in settings with a high prevalence of this condition, a daily intake of 10–12.5 mg of elemental iron is recommended for children aged 6–23 months, and 30 mg/day for those aged 24–59 months, administered for a period of three months. In children with anemia, treatment consists of therapeutic doses of 3–6 mg/kg/day until Hb levels normalize, followed by an additional period of supplementation to replenish iron stores, in accordance with national guidelines [29].

In this study, for infants without anemia at baseline, the prescribed dose was 3 mg/kg/day (maximum 40 mg/day). Infants with mild or moderate anemia received 3 mg/kg/day with a higher maximum dose (70 mg/day) as therapeutic iron in the form of ferrous sulfate for six months [15].

The infants without anemia received 30 sachets per month for a period of six months. Each 1 g sachet of MNP contained 12.5 mg of elemental iron, 5 mg of zinc, 160 µg of folic acid, 300 µg of vitamin A (retinol equivalents), and 30 mg of vitamin C, and was added to a small portion of the infant’s food. Children with anemia received iron in syrup according to guidelines from MINSA [13].

Strict follow-up consisted of a policy-mandated enhanced supervision framework implemented for six months, regardless of baseline anemia status, and delivered by a multidisciplinary healthcare team. This strategy comprised multiple components delivered as part of routine public health care, including adherence support, nutritional counselling, reminder calls, home visits, and preventive interventions. Therefore, the exposure evaluated in this study reflects an enhanced follow-up package rather than supervision alone.

The follow-up strategy focused on adherence support and monitoring through monthly clinic visits, reinforcement of dietary and micronutrient recommendations, reminder telephone calls, and up to three home visits during the follow-up period. Adherence to MNP or therapeutic iron supplementation was assessed during these contacts through caregiver self-report and routine verification of supplement use as part of standard public health practice. However, no objective adherence measures, such as sachet counts, direct observation, or biochemical markers, were available in this programmatic setting.

As part of routine public health care, infants under strict follow-up also received a single prophylactic dose of mebendazole (500 mg).

Adverse effects were recorded based on caregiver reports during clinic visits and telephone contacts. In contrast, infants in the non-strict follow-up group received MNP or therapeutic iron but did not participate in a structured supervision or adherence support program. Importantly, the strict follow-up framework did not modify iron dose, formulation, or duration, and differed exclusively in monitoring and adherence support intensity.

**Diagnosis of anemia:** Anemia was defined using altitude-adjusted Hb values following 2024 WHO recommendations, which apply a correction factor of 2.6 g/dL for the altitude of 3832 meters [8]. Hb concentration was measured with a portable hemoglobinometer (HemoCue Hb 201 + ; HemoCue AB, Ängelholm, Sweden) using capillary blood obtained by finger puncture of the ring or index finger, the second drop of blood was used to minimize contamination with interstitial fluid. Equipment was calibrated, and measurements were performed by trained laboratory technicians at the Health Facility at baseline and after 6 months of supplementation. Each infant therefore had two recorded Hb values (i.e., baseline on the 6-month visit and again on the 12-month visit).

**Nutritional status:** Anthropometric measurements were taken at the start of recruitment and at month six of follow-up by qualified healthcare personnel using calibrated scales and infantometers.

Nutritional status was assessed using Z-scores for weight for height (WHZ), weight for age (WAZ), and height for age (HAZ). Z-scores were calculated at baseline and at the end of follow-up using the WHO Child Growth Standards (2006) and the WHO Anthro methodology [30]. Individual Z-scores were used for all subsequent statistical analyses.

**Additional information:** Additional information on access to basic services, maternal education level, and type of feeding prior to 6 months of age was obtained from medical records.

**Outcome:** The primary outcome was adequate hemoglobin response, defined as an increase of ≥1 g/dL after six months of supplementation [31–35]. Secondary outcomes included change in hemoglobin concentration as a continuous variable (ΔHb), anemia status at six months of follow-up, patterns of hematological status after supplementation (worsening, persistence, maintenance of normal Hb, or reversal of anemia), and reported adverse effects during supplementation (Fig 1).

**Bias**: Selection bias was minimized by including nearly all eligible infants attending routine growth and development visits during the study periods. Assignment to strict or non-strict follow-up was determined solely by the timing of policy implementation, resulting in exposure groups defined by the timing of policy implementation.

Hb and anthropometric measurements were conducted using standardized procedures and calibrated equipment, thereby limiting information bias. However, residual confounding due to unmeasured secular changes between periods cannot be fully excluded. In addition, the higher reporting of adverse events under strict follow-up likely reflects enhanced surveillance rather than increased toxicity, introducing potential detection bias. Confounding was addressed through multivariable adjustment and sensitivity analyses.

During the study period, no major changes in local nutritional programs, infant feeding recommendations, food supplementation policies, or healthcare access were identified at the El Vallecito Health Facility beyond the implementation of the strict supervision framework.

Both cohorts were recruited from the same catchment population under the same routine public health infrastructure. In addition, recruitment periods covered complete annual cycles, which likely reduced the influence of seasonal variation in food availability or dietary practices.

This study is reported in accordance with the Strengthening the Reporting of Observational Studies in Epidemiology (STROBE) guidelines for cohort studies. A completed STROBE checklist is provided as Supplementary Table 1.

**Statistical analyses:** Continuous variables were assessed for normality using the Shapiro–Wilk test and visual inspection. As several variables were non-normally distributed, baseline comparisons across groups defined by follow-up condition (strict vs non-strict) and baseline anemia status (anemic vs non-anemic) were performed using the Kruskal–Wallis’s test, with Dunn’s post-hoc tests and Bonferroni correction for clinically relevant contrasts. Categorical variables were compared using Pearson’s chi-square or Fisher’s exact tests, as appropriate, excluding missing values from denominators.

Hb outcomes were analyzed using complementary approaches. Hb response (ΔHb ≥ 1 g/dL) was evaluated using logistic regression. Change in Hb over follow-up (ΔHb) was analyzed as a continuous outcome using multivariable linear regression. Anemia prevalence at six months was compared descriptively between follow-up groups using chi-square tests.

Models were adjusted for age, sex, and baseline anemia status, with covariates selected based on clinical relevance and bivariable associations (p < 0.20).

Total iron received was excluded from main models as a potential mediator and included in sensitivity analyses. Effect modification was explored using interaction terms between follow-up condition, baseline anemia status, and baseline age. Model assumptions were assessed using residual diagnostics and variance inflation factors. Analyses were conducted using Stata 18.0, with a two-sided p value <0.05 considered statistically significant.

**Ethical considerations:** Ethical approval was obtained from the Regional Health Directorate of Puno (Certificate N°. 012–2020-Red Puno). The study consisted of a secondary analysis of routinely collected, anonymized clinical and programmatic data. Because only anonymized secondary data were analyzed, individual informed consent was not required. The study was also reviewed and approved by the Institutional Ethics Committee of the University of San Ignacio de Loyola (Code No. 2025076, Certificate N^°^. 2026−001).

## Results

### Baseline characteristics and anemia prevalence

Infants enrolled in the study showed comparable anthropometric, hematological, and perinatal characteristics across groups defined by follow-up intensity and anemia status. As expected, altitude-adjusted Hb concentrations differentiated anemic from non-anemic infants at baseline, while observed Hb values followed the same pattern. Baseline perinatal characteristics did not show relevant differences between subgroups (Table 1).

**Table 1:** Characteristics at baseline of infants, and mothers in the highlands of El Vallecito, Puno (3832 m.a.s.l) according to follow-up conditions.

Characteristics	Non strict follow-up (Total n=178)		Strict follow-up (Total n=139)	
	Non anemic (n=113)	Anemic (n=65)	Non anemic (n=97)	Anemic (n=42)
Adjusted Hb (g/dL)^*^	11.2±0.5	9.7±0.6	11.5±0.7	9.8±0.6
Observed Hb (g/dl)	13.8 ± 0.5	12.3 ± 0.6	14.5 ± 0.7	12.4 ± 0.6
Number of pregnancies	1.6 ± 0.8	2.0 ± 1.4	1.8± 1.1	1.9 ± 1.0
Gestational age at birth (weeks)	39.5±1.9	38. 4±1.9	39.0±1.2	38.6±1.2
Height at birth (cm)	50.2 ± 1.7	49.5 ± 2.7	49.9 ± 1.5	49.3 ± 1.4
Birth weight (grams)	3348±385	3157±485	3258±346	3150±367
Cephalic perimeter (cm)	34.6 ± 1.3	34.5 ± 2.7	34.6 ± 1.7	34.2 ± 1.7
Apgar 1 minute	7.9 ± 0.9	7.6 ± 1.6	7.9 ± 0.7	7.7 ± 1.2
Apgar 5 minutes	9.0 ± 0.4	8.9 ± 0.8	9.1 ± 0.4	8.8 ± 0.8

Data are expressed as means ± standard deviation.

* Hemoglobin values were adjusted for altitude according to the new WHO guidelines [8].

Sociodemographic characteristics were comparable between children included in the strict and non-strict follow-up groups. Prenatal, perinatal, and early postnatal clinical characteristics were largely comparable between children enrolled in strict and non-strict follow-up programs (Supplementary Table 2).

Program implementation indicators differed markedly between follow-up strategies. Children in the strict follow-up group were more likely to undergo parasitological examination, receive antiparasitic prophylaxis, and benefit from more intensive nutritional counseling and home visits (all χ² tests, p < 0.001). In contrast, biological outcomes were largely similar, including the prevalence of intestinal parasites and early feeding practices (Supplementary Table 2).

### Anthropometry by follow-up condition and baseline anemia

Infants were classified into four groups according to follow-up condition (strict vs. non-strict) and baseline anemia status (anemic vs. non-anemic). Age did not differ across groups. At baseline, overall differences were observed for body weight and length across the four groups (Kruskal–Wallis p = 0.0106 and p = 0.0198, respectively), with lower values among infants with baseline anemia. However, none of the planned post-hoc pairwise comparisons remained statistically significant after Bonferroni correction. Overall, growth patterns over the 6-month follow-up were broadly similar across follow-up conditions and anemia strata.

When comparing strict versus non-strict follow-up, baseline anemia indicators, both unadjusted and altitude-adjusted, were comparable between groups. At 6 months, unadjusted anemia prevalence did not differ significantly; however, altitude-adjusted anemia was significantly lower in the strict follow-up group (χ², p = 0.019).

The distribution of hematological status after MNP or therapeutic iron supplementation, including worsening, lack of improvement, maintenance of normal Hb, and reversal of anemia, did not differ significantly between follow-up groups (χ², p = 0.27). Hb response (ΔHb ≥ 1 g/dL) was also similar between groups. In contrast, reported adverse effects related to MNP or therapeutic iron supplementation were significantly more frequent in the strict follow-up group (p < 0.001) (Table 2).

**Table 2:** Anemia-related categorical outcomes and supplementation effects according to follow-up condition (Strict vs non-strict).

Variable	Strict follow-up (n = 139)	Non Strict follow-up (n = 178)	p-value*
Sex			
Male Female	75 64	99 79	0.86
Baseline anemia			
No Yes	138 1	175 3	0.80
Adjusted baseline anemia			
No Yes	97 42	113 65	0.29
Anemia at month 6			
No Yes	139 0	174 4	0.20
Adjusted anemia at month 6			
No Yes	104 35	110 68	0.019
Status after supplementation			
Worsened Anemia did not improve Maintained as normal Reversed anemia	12 23 85 19	31 37 82 28	0.27
Hb response to MNP or therapeutic iron supplementation			
No Yes	113 26	157 21	0.12
Adverse effects to MNP or therapeutic iron supplementation			
No Yes	85 54	175 3	<0.001

### Hemoglobin response and anemia status after supplementation

**Fig 2:**
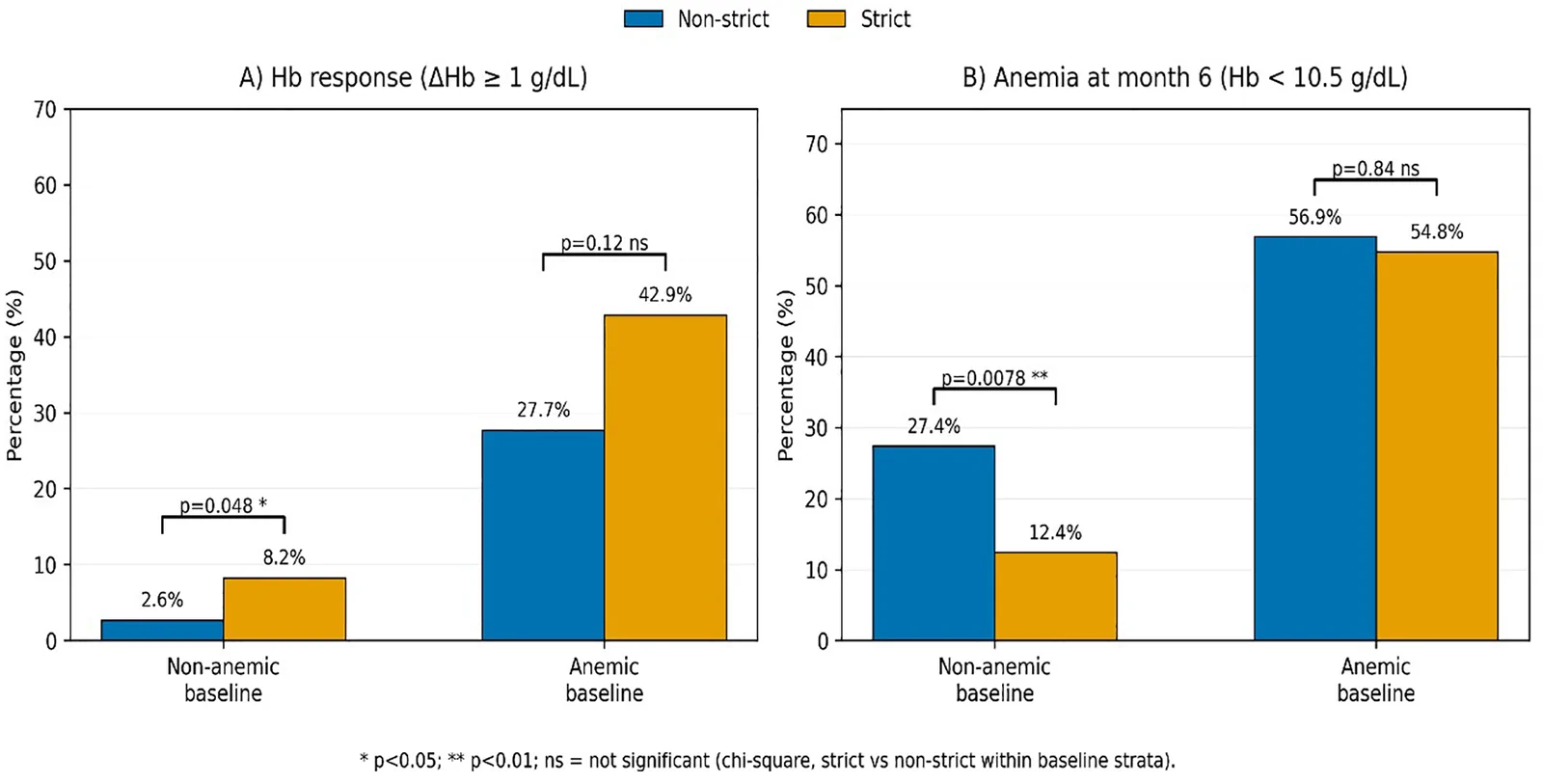
Hemoglobin response and anemia outcomes after 6 months of MNP or therapeutic iron supplementation according to baseline anemia status and follow-up intensity in high-altitude infants. Anemia was defined according to WHO 2024 criteria. p-values: chi-square tests. **Left.** Proportion of infants achieving a hemoglobin increase ≥1 g/dL after six months of MNP or therapeutic iron supplementation, stratified by baseline anemia status and follow-up condition (strict vs non-strict). **Right.** Prevalence of anemia (altitude-adjusted Hb < 10.5 g/dL) after six months of MNP or therapeutic iron supplementation according to baseline anemia status and follow-up condition.

Overall, 14.8% of infants achieved a ΔHb ≥ 1 g/dL after six months of MNP or therapeutic iron supplementation. The proportion of responders was higher in the strict follow-up group than in the non-strict follow-up group (18.7% vs. 11.8%); however, this difference did not reach statistical significance in the unadjusted comparison (χ² = 2.43, p = 0.12) (Fig 2A).

Hb response differed according to baseline anemia status. Among non-anemic infants at baseline, a significantly higher proportion achieved a ΔHb ≥ 1 g/dL under strict follow-up compared with non-strict follow-up (8.3% vs. 2.7%, p = 0.048). Among infants with baseline anemia, response rates were higher overall, with a greater proportion of responders in the strict follow-up group, although the difference was not statistically significant (p = 0.12).

In both follow-up strategies, hematologic response was strongly influenced by baseline anemia status, while strict follow-up appeared to provide additional benefit, particularly among infants who were non-anemic at baseline (Fig 2A).

The prevalence of anemia at six months also differed according to baseline anemia status and follow-up condition (Fig 2B). Among infants who were non-anemic at baseline, anemia prevalence at follow-up was significantly lower in the strict follow-up group than in the non-strict follow-up group (12.4% vs. 27.4%, p = 0.008). Overall, 43 of 210 infants (20.4%) initially classified as non-anemic developed anemia after six months of supplementation.

Among infants with baseline anemia, anemia prevalence at six months remained high and did not differ significantly between strict and non-strict follow-up conditions. In both strategies, these infants had a substantially higher prevalence of anemia at follow-up than those who were non-anemic at baseline (p < 0.001). These findings indicate that baseline anemia was the principal determinant of persistent anemia, whereas strict follow-up was associated with a lower prevalence of anemia among infants who were non-anemic at baseline (Fig 2B).

### Age-related hemoglobin change over follow-up

**Fig 3:**
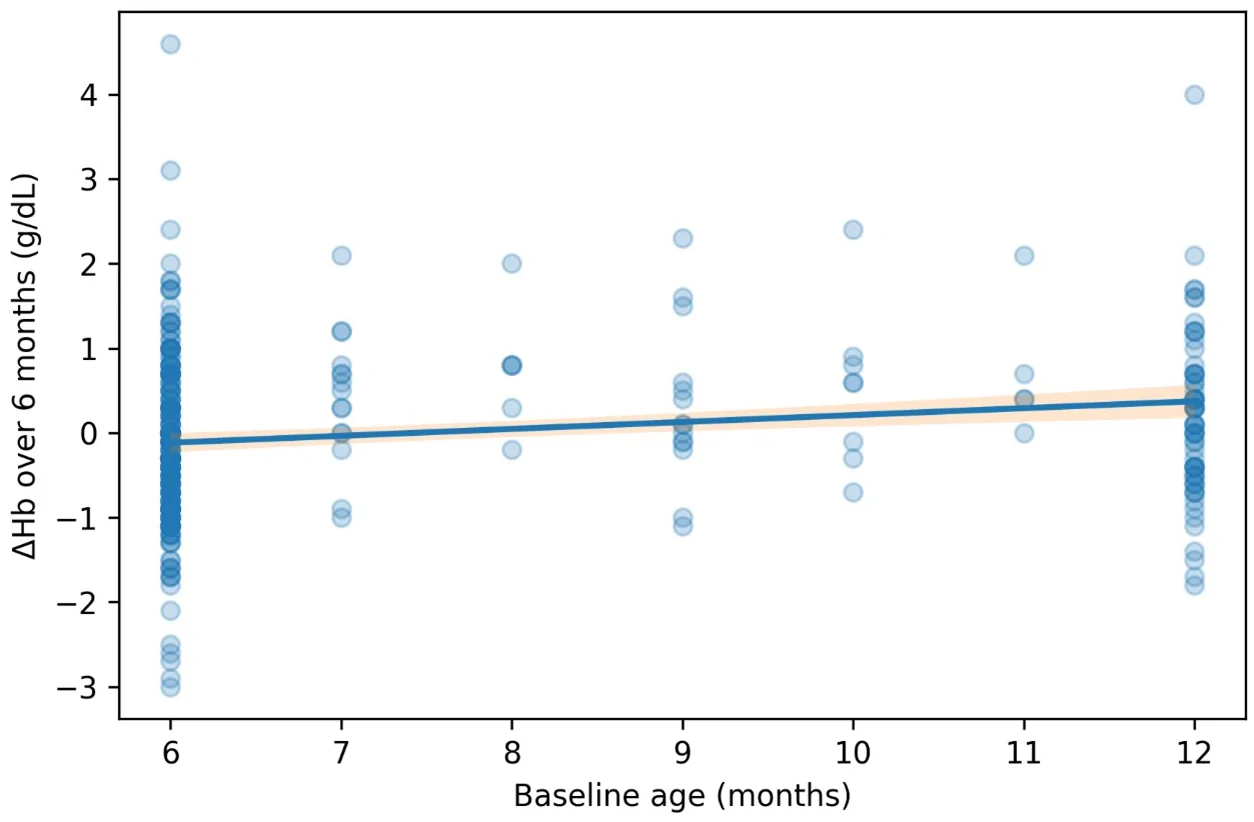
Multivariable-adjusted change in hemoglobin over 6 months according to baseline age in infants living at high altitude. Predicted values of hemoglobin change over follow-up (ΔHb = final − baseline). Multivariable linear regression model adjusted for baseline anemia status, sex, and total iron received.

Using Hb change over follow-up as a continuous outcome (ΔHb = final − baseline), baseline age was independently associated with an increase in Hb over the 6-month supplementation period. In multivariable linear regression analyses adjusted for baseline anemia status, sex, and total iron received, each additional month of age at study entry was associated with an approximately 0.08 g/dL greater increase in Hb (p < 0.001) (Fig 3).

### Multivariable regression models

Hemoglobin response ≥1 g/dL was more frequent among infants receiving strict follow-up than among those under routine surveillance. Although the unadjusted comparison of response proportions did not reach statistical significance (18.7% vs. 11.8%; p = 0.12), multivariable analyses were performed because hemoglobin response was strongly influenced by baseline anemia status and other participant characteristics. Similar analyses were performed using final anemia status as the outcome variable, yielding comparable results (Supplementary Table 3).

In the crude logistic regression model, strict follow-up was associated with higher odds of achieving an adequate hemoglobin response (OR 2.45; 95% CI 1.30–4.63; p = 0.005). This association remained significant in the prespecified primary adjusted model, which included baseline anemia status, age, and sex (aOR 3.53; 95% CI 1.40–8.84; p = 0.007).

In the fully adjusted sensitivity model, which additionally included maternal education, infant feeding history, drinking water access, birth weight, and total iron received, the association was attenuated and no longer reached statistical significance (aOR 2.18; 95% CI 0.53–8.99; p = 0.280).

Birth weight was independently associated with hemoglobin response in the fully adjusted model (p = 0.016), whereas baseline anemia status, age, sex, maternal education, feeding practices, drinking water access, and total iron received were not significantly associated with the outcome (Table 3).

**Table 3:** Multivariable logistic regression for hemoglobin response (ΔHb ≥ 1 g/dL).

Hemoglobin response ≥1 g/dL (dichotomous)	Model 1			Model 2			Model 3		
	cOR	CI 95%	p-value	aOR	CI 95%	p-value	aOR	CI 95%	p-value
Strict follow-up									
No	Ref.			Ref.			Ref.		
Yes	2.45	1.30-4.63	0.005	3.53	1.40-8.84	0.007	2.18	0.53-8.99	0.28
Anemia basal									
No	Ref.			Ref.			Ref.		
Yes	2.37	0.24-23.30	0.459	6.47	0.28-147.53	0.242	6.12	0.25-14.73	0.264
Total iron	1.00	1.00-1.00	0.014	-	-	-	1.00	0.99-1.09	0.374
Basal age (months)	1.01	0.96-1.07	0.566	1.01	0.94-1.09	0.663	1.01	0.93-1.09	0.832
Sex									
Female	Ref			Ref.			Ref.		
Male	1.32	0.69-2.52	0.388	1.75	0.76-4.03	0.186	1.76	0.76-4.06	0.183
Mother education									
Elementary	Ref.			Ref.			Ref.		
High School	1.51	0.38-5.83	0.553	1.98	0.43-9.01	0.377	2.12	0.45-9.89	0.339
Tecnician	2.19	0.39-5.83	0.367	1.47	0.19-11.49	0.709	1.66	0.21-13.35	0.632
University	1.79	0.49-6.40	0.373	2.81	0.68-11.62	0.153	2.87	0.68-12.09	0.150
Nutrition									
Exclusive breastfeeding	Ref.			Ref.			Ref.		
Mixed	1.41	0.50-3.92	0.510	0.41	0.09-1.81	0.245	0.43	0.09-1.88	0.262
Drinking water									
No	Ref.			Ref.			Ref.		
Yes	0.28	0.02-3.22	0.310	0.15	0.01-2.07	0.161	0.16	0.01-2.06	0.160
Birth weigth (g)	0.99	0.99-1.00	0.103	0.99	0.99-0.99	0.021	0.99	0.99-0.99	0.016

cOR: crude Odds Ratio, aOR: adjusted Odds Ratio. Odds ratios (OR) and 95% confidence intervals (CI).

Model 1: Unadjusted analysis of the relationship between the exposure variables and the outcome variable.

Model 2: Adjusted analysis without considering total iron intake.

Model 3: Adjusted analysis considering total iron intake.

In the linear regression analyses evaluating change in hemoglobin concentration between baseline and follow-up, infants receiving strict follow-up showed greater increases in hemoglobin compared with those under routine surveillance. In the crude model, strict follow-up was associated with a mean hemoglobin increase of 0.49 g/dL (95% CI 0.29–0.70; p < 0.001). This association remained significant after adjustment for baseline anemia status, age, and sex (aCoef 0.67 g/dL; 95% CI 0.41–0.93; p < 0.001), and after additional adjustment for maternal education, feeding history, drinking water access, birth weight, and total iron received (aCoef 0.74 g/dL; 95% CI 0.23–1.25; p = 0.005).

Baseline anemia status was independently associated with greater hemoglobin gains in adjusted models (aCoef 1.35 g/dL; 95% CI 0.02–2.68; p = 0.046). In the model additionally adjusted for baseline hemoglobin concentration, the effect of strict follow-up was attenuated and no longer reached statistical significance (aCoef 0.40 g/dL; 95% CI −0.05 to 0.84; p = 0.079), while baseline hemoglobin showed a strong inverse association with hemoglobin change (aCoef −0.56 g/dL; 95% CI −0.68 to −0.43; p < 0.001). Older age was associated with greater hemoglobin increases (aCoef 0.03 g/dL per month; 95% CI 0.01–0.06; p = 0.007), whereas lower birth weight remained associated with larger hemoglobin changes across models (Table 4).

**Table 4:** Multivariate linear regression for changes in hemoglobin at six months (end-to-baseline).

Change in hemoglobin (final-basal)	Model 1			Model 2			Model 3			Model 4		
	cCoef	CI 95%	p-value	aCoef	CI 95%	p-value	aCoef	CI 95%	p-value	aCoef	CI 95%	p-value
Strict follow-up												
No	Ref.			Ref.			Ref.			Ref.		
Yes	0.49	0.29-0.70	<0.001	0.67	0.41-0.93	<0.001	0.74	0.23-1.25	0.005	0.40	−0.05-0.84	0.079
Anemia basal												
No	Ref.			Ref.			Ref.			–	–	–
Yes	0.52	−0.44-1.49	0.290	1.35	0.02-2.68	0.046	1.36	0.02-2.69	0.045	–	–	–
Total iron	<0.01	0.01-0.01	<0.001	–	–	–	<−0.01	−0.01-0.01	0.774	<−0.01	−0.01-0.01	0.59
Basal Hb (g/dL)				–	–	–	–	–	–	−0.56	−0.68--0.43	<0.001
Basal age (months)	0.01	−0.01-0.03	0.228	0.01	−0.02-0.04	0.428	0.01	−0.01-0.04	0.400	0.03	0.01-0.06	0.007
Sex												
Female	Ref			Ref.			Ref.			Ref.		
Male	−0.07	−0.28-0.13	0.477	0.10	−0.15-0.35	0.434	0.10	−0.15-0.35	0.435	0.08	−0.13-0.30	0.438
Mother education												
Elementary	Ref.			Ref.			Ref.			Ref.		
High School	−0.04	−0.43-0.34	0.818	0.05	−0.33-0.45	0.771	0.05	−0.34-0.45	0.789	−0.08	−0.42-0.26	0.638
Tecnician	0.08	−0.47-0.65	0.758	−0.09	−0.66-0.48	0.759	−0.10	−0.68-0.48	0.740	−0.20	−0.70-0.30	0.431
University	0.08	−0.27-0.45	0.635	0.14	−0.22-0.52	0.426	0.15	−0.21-0.52	0.423	0.08	−0.23-0.40	0.599
Nutrition												
Exclusive breastfeeding	Ref.			Ref.			Ref.			Ref.		
Mixed	0.24	−0.12-0.61	0.197	−0.06	−0.47-0.33	0.743	−0.07	−0.48-0.34	0.733	0.17	−0.18-0.52	0.349
Drinking water												
No	Ref.			Ref.			Ref.			Ref.		
Yes	−0.38	−1.53-0.76	0.515	−0.44	−1.53-0.64	0.426	−0.44	−1.53-0.65	0.425	−0.23	−1.17-0.71	0.635
Birth weigth (g)	−0.01	−0.01--0.01	<0.001	−0.01	−0.01--0.01	<0.001	−0.01	−0.01--0.01	<0.001	−0.01	−0.01--0.01	0.019

cOR: crude Odds Ratio, aOR: adjusted Odds Ratio. Odds ratios (OR) and 95% confidence intervals (CI).

Model 1: Unadjusted analysis of the relationship between the exposure variables and the outcome variable. Model 2: Adjusted analysis without considering total iron intake.

Model 3: Adjusted analysis considering total iron intake. Model 4: adjusted analysis accounting for sensitivity in the group of patients with anemia at baseline.

## Discussion

In this policy-driven natural experiment among infants living at high altitude (HA), strict follow-up was associated with improved hemoglobin outcomes and a lower prevalence of altitude-adjusted anemia at follow-up. The most consistent finding was observed when hemoglobin was analyzed as a continuous outcome, with infants receiving strict follow-up experiencing greater increases in Hb concentration over the six-month supplementation period, even after adjustment for demographic, socioeconomic, nutritional, and program-related factors. These findings suggest that enhanced follow-up may contribute to modest but measurable improvements in hemoglobin status under routine programmatic conditions. In contrast, although strict follow-up was associated with higher odds of achieving the prespecified hematologic response threshold of an Hb increase ≥1 g/dL in the primary adjusted model, this association was attenuated and no longer statistically significant in the fully adjusted sensitivity analysis. Notably, baseline anemia status was a stronger predictor of hematologic response than follow-up intensity itself, and a substantial proportion of infants remained anemic after six months of supplementation [28,31–35].

In this study, changes in hemoglobin analyzed as a continuous outcome appeared more sensitive for detecting intervention-associated differences than the dichotomous threshold of Hb increase ≥1 g/dL.

One possible explanation for the limited effectiveness of supplementation relates to the diagnosis of anemia at HA. Evidence from South America, Africa, and Asia indicates that anemia prevalence may be overestimated when Hb-based definitions and altitude-adjustment schemes do not adequately account for physiological and genetic adaptation [18–20].

Despite the revised WHO altitude-adjustment criteria introduced in 2024, uncertainty remains regarding the optimal classification of anemia at HA [8,36]. Comparisons between genetically adapted and non-adapted populations, such as Tibetans and Han migrants residing at similar altitudes, further suggest that lower Hb concentrations may reflect adaptation rather than pathology [37,38].

A second explanation is that anemia in this population may be multifactorial and therefore not fully responsive to iron-based interventions alone. Potential contributors include inflammation-related anemia, recurrent infections, parasitic disease, environmental enteric dysfunction, nutritional deficiencies, and hemoglobinopathies. These mechanisms could not be evaluated directly because biomarkers of iron status, inflammation, and hemoglobinopathies were not available.

Inflammatory pathways may also impair iron absorption and mobilization through hepcidin-mediated mechanisms, reducing hematologic response despite adequate micronutrient intake.

Consistent with this hypothesis, approximately half of infants with baseline anemia remained anemic after six months of supplementation, even under strict supervision. Similar findings have been reported in southern Peru, where children exposed and unexposed to MNP showed comparable iron status despite marked differences in anemia prevalence when altitude-adjusted Hb thresholds were applied [39,40].

While MNP and therapeutic iron remain important interventions for preventing and treating nutritional deficiencies [41], these findings suggest that universal supplementation alone may be insufficient and support the need for more context-specific approaches to anemia control in HA populations.

An additional finding was the strong association between age and Hb change during follow-up. Hb increased, and anemia prevalence declined with advancing age independent of supplementation intensity, reflecting physiological maturation and the transition from fetal to adult hemoglobin [7].

This observation suggests that physiological maturation contributes importantly to hemoglobin dynamics during infancy and should be considered when interpreting hematologic responses to supplementation.

The occurrence of incident anemia among infants initially classified as non-anemic, also described in other Peruvian studies [42], further highlights the dynamic nature of Hb during infancy and the limitations of applying static diagnostic thresholds during a period of rapid physiological change. Beyond developmental factors, non–iron-deficiency mechanisms may contribute to the modest hematologic response observed.

In iron-replete infants, excess iron exposure may promote low-grade inflammation, alter gut microbiota composition, and increase hepcidin activity, leading to functional anemia that is less responsive to supplementation [43,44]. Although these mechanisms were not evaluated directly, they are biologically plausible and supported by experimental and epidemiological evidence.

HA populations present unique challenges for anemia research and clinical practice. Andean populations exhibit larger increases in Hb with altitude than most other populations worldwide, whereas altitude-related Hb changes are smaller in children than in adults [37,45,46]. This heterogeneity raises questions about the universal application of a single altitude-correction scheme across diverse populations and age groups.

At the population level, the limited effectiveness of MNP or therapeutic iron programs observed in this study is consistent with national data from Peru, where increased program coverage has not led to substantial reductions in the prevalence of childhood anemia [47].

The absence of differences in anthropometric outcomes between follow-up strategies further suggests that intensified iron or MNP supplementation alone may have limited effects on broader nutritional outcomes during late infancy. The low hematologic response observed in this cohort should therefore be interpreted as supporting a hypothesis of multifactorial anemia rather than directly excluding iron deficiency as the predominant cause.

These findings are also consistent with randomized evidence showing that improvements in iron status do not necessarily translate into proportional gains in broader developmental outcomes during early childhood [48].

Reported adverse effects were more frequent in the strict follow-up group, likely reflecting more complete detection and reporting due to enhanced surveillance and more frequent contact with caregivers rather than a true increase in iron-related toxicity.

### Strengths and limitations

This study has several strengths. First, it was conducted within a real-world public health program implemented under routine conditions, providing evidence that is highly relevant for policy and clinical practice. Second, the policy-driven natural experiment allowed evaluation of an enhanced follow-up strategy in a context where randomized trials are not feasible because universal iron supplementation is mandated by national guidelines. Third, the study included near-complete capture of eligible infants from a well-defined high-altitude population through the national nominal registry system, minimizing selection bias. Finally, The simultaneous evaluation of categorical and continuous hemoglobin outcomes allowed a more comprehensive assessment of hematologic response. Notably, continuous hemoglobin change provided a more sensitive measure of intervention-associated differences.

This study also has important limitations. First, the before-and-after observational design is susceptible to residual confounding from unmeasured secular or seasonal changes. Second, adherence to MNP or therapeutic iron supplementation was not assessed using objective measures such as sachet counts, direct observation, pharmacy refill records, or biochemical markers. Third, the enhanced follow-up strategy represented a bundled intervention that included nutritional counselling, reminder calls, home visits, parasitological evaluation, and mebendazole prophylaxis, preventing identification of the individual contribution of each component. Fourth, biomarkers of iron status, inflammation, folate and vitamin B12 status, and hemoglobinopathies were not available, limiting etiological interpretation of anemia persistence and hematologic response. Fifth, anemia was defined using hemoglobin-based criteria alone, which may not fully capture iron status in high-altitude populations. Sixth, adverse effects may have been differentially detected because of more intensive surveillance in the strict follow-up group. Finally, the study was conducted in a single high-altitude district, which may limit the generalizability of the findings to other populations.

## Conclusions

This policy-driven natural experiment demonstrates that strict follow-up of MNP or therapeutic iron supplementation was associated with improved hemoglobin outcomes and a lower prevalence of altitude-adjusted anemia at follow-up among infants living at high altitude. These findings suggest that enhanced follow-up may improve hematologic outcomes under routine public health conditions, although anemia persistence indicates that additional biological, environmental, and nutritional factors likely contribute to anemia in this setting.

Data are presented as n. Comparisons between strict and non-strict follow-up *p-value: Pearson’s chi-square test or Fisher’s exact test. Anemia was defined using both unadjusted Hb values and Hb adjusted for altitude according to WHO criteria [8]. Response to MNP or therapeutic iron supplementation was defined as an ΔHb ≥ 1 g/dL at 6 months.

## Supporting information

S1 Table STROBE Statement”. (DOCX)

S2 Table Characteristics at baseline of infants, mothers, and sanitary conditions in the highlands of El Vallecito, Puno (3832 m.a.s.l)”. This is the S2 Table legend “#Data expressed as means + standard deviation. The rest of the data are relative frequencies (%). p < 0.05, Student’s t-test between anemic and non-anemic infants within the same group.
(DOCX)

S3 Table Multivariable logistic regression for final anemia diagnosis”. This is the S3 Table legend “cOR: crude Odds Ratio, aOR: adjusted Odds Ratio. Odds ratios (OR) and 95% confidence intervals (CI). Model 1: Unadjusted analysis of the relationship between the exposure variables and the outcome variable. Model 2: Adjusted analysis.”.
(DOCX)
