# Supplementary material for: Hemoglobin outcomes associated with supervised therapeutic iron or preventive multiple micronutrient powder supplementation in high-altitude Peruvian infants: A real-world natural experiment
Source: PLoS One. 2026 Aug 26;21(8):e0356793. doi: 10.1371/journal.pone.0356793 (PMC13514055; doi:10.1371/journal.pone.0356793)
Supplement: Supplementary file 2 [file pone.0356793.s002.docx]

**Supplementary Table 2.** Characteristics at baseline of infants, mothers, and sanitary conditions in the highlands of El Vallecito, Puno (3832 m.a.s.l).

|  | **Not strict follow-up** | | **Strict follow-up** | | |  |
| --- | --- | --- | --- | --- | --- | --- |
| **Characteristic** | **Not anemic**  **(n=121)** | **Anemic**  **(n=52)** | **Not anemic**  **(n=100)** | | **Anemic**  **(n=36)** |  |
| Weight in Kg | 8.57 ± 1.13 | 8.38 ± 1.43 | 8.38 ±1.14 | 8.01 ± 0.90 | |  |
| Height in cm | 69.01 ± 4.12 | 68.40 ± 4.20 | 68.28 ± 3.83 | 67.44 ± 4.41 | |  |
| Adjusted Hb in g/dL | 11.23 ± 0.54 | 9.82 ± 0.52* | 11.47 ± 0.70 | 9.77 ± 0.55* | |  |
| Weight-for-lenght-Z-score | 0.64 ± 0.97 | 0.56 ± 1.13 | 0.64 ± 0.92 | 0.77 ± 1.26 | |  |
| Length-for-age-Z-score | -0.24 ± 1.16 | -0.38 ± 1.31 | -0.34 ± 0.94 | -0.40 ± 1.84 | |  |
| Weight-for-age-Z-score | 0.30 ± 0.99 | 0.09 ± 1.18 | 0.24 ± 0.89 | -0.01 ± 0.89 | |  |
| Gestational age at birth in weeks | 38.90 ± 4.90 | 38.85 ± 0.97 | 39.04 ± 1.17 | 38.52 ± 1.27 | |  |
| Birth weight | 3347.38 ± 379.73 | 3248.07 ± 326.01 | 3266.33 ± 328.08 | 3179.72 ± 359.10 | |  |
| Sex  Female | 52 (42.98) | 25 (48.08) | 47 (47.00) 16 (44.44) | | |  |
| Mother education  Elementary  High school  Tecnician  University | 4 (3.31)  32 (26.45)  4 (3.31)  35 (28.93) | 2 (3.85)  13 (25.00)  0 (0.00)  13 (25.00) | 8 (8.00) 4 (11.11)  28 (28.00) 9 (25.00)  6 (6.00) 4 (11.11)  52 (52.00) 15 (41.67) | | |  |
| Access to piped water  Yes | 61 (50.41) | 26 (50.00) | 96 (96.00) 34 (94.44) | | |  |
| Sewage at home  Yes | 61 (50.41) | 25 (48.08) | 89 (89.00) 33 (91.67) | | |  |
| Data of nutrition at 6 months  Exclusive breastfeeding  Artificial milk only  Both | 83 (68.60)  5 (4.13)  33 (27.27) | 35 (67.31)  1 (1.92)  16 (30.77) | 83 (83.00) 32 (88.84)  1 (1.00) 0 (0.00)  15 (15.00) 3 (8.33) | | |  |

^#^Data expressed as means + standard deviation. The rest of the data are relative frequencies (%).

*p* < 0.05, Student’s *t*-test between anemic and non-anemic infants within the same group.
