# Supplementary material for: Hemoglobin outcomes associated with supervised therapeutic iron or preventive multiple micronutrient powder supplementation in high-altitude Peruvian infants: A real-world natural experiment
Source: PLoS One. 2026 Aug 26;21(8):e0356793. doi: 10.1371/journal.pone.0356793 (PMC13514055; doi:10.1371/journal.pone.0356793)
Supplement: Supplementary file 3 [file pone.0356793.s003.docx]

**Supplementary Table 3.** Multivariable logistic regression for final anemia diagnosis.

| **Final anemia diagnosis (dichotomous)** | | **Model 1** | | | **Model 2** | | |
| --- | --- | --- | --- | --- | --- | --- | --- |
|  |  | **cOR** | **CI 95%** | **p-value** | **aOR** | **CI 95%** | **p-value** |
| Strict follow-up | |  |  |  |  |  |  |
|  | No | Ref. |  |  | Ref. |  |  |
|  | Yes | 2.81 | 1.63-4.82 | <0.001 | 10.06 | 2.21-45.70 | 0.003 |
| Total iron | | 1.00 | 1.00-1.00 | 0.015 | 0.99 | 0.99-1.00 | 0.833 |
| Basal age (months) | | 0.96 | 0.92-1.00 | 0.081 | 0.92 | 0.85-0.97 | 0.009 |
| Sex | |  |  |  |  |  |  |
|  | Female | Ref |  |  | Ref. |  |  |
|  | Male | 0.76 | 0.47-1.23 | 0.271 | 0.70 | 0.37-1.29 | 0.256 |
| Mother education | |  |  |  |  |  |  |
|  | Elementary | Ref. |  |  | Ref. |  |  |
|  | High School | 1.87 | 0.81-4.32 | 0.138 | 1.88 | 0.73-4.84 | 0.186 |
|  | Tecnician | 3.18 | 0.77-13.02 | 0.107 | 1.67 | 0.34-8.12 | 0.527 |
|  | University | 1.47 | 0.68-3.16 | 0.319 | 1.28 | 0.54-3.03 | 0.572 |
| Nutrition | |  |  |  |  |  |  |
|  | Exclusive breastfeeding | Ref. |  |  | Ref. |  |  |
|  | Mixed | 0.84 | 0.38-1.87 | 0.682 | 0.63 | 0.24-1.63 | 0.345 |
| Birth weigth (g) | | 0.99 | 0.99-1.00 | 0.020 | 0.99 | 0.99-1.00 | 0.170 |

cOR: crude Odds Ratio, aOR: adjusted Odds Ratio. Odds ratios (OR) and 95% confidence intervals (CI).

Model 1: Unadjusted analysis of the relationship between the exposure variables and the outcome variable.

Model 2: Adjusted analysis.
